# Inside the Fire. Exploring the Impact of Anxiety, Depression, and Sleep Disturbances on Pain Perception in Burning Mouth Syndrome: A Cross‐Sectional Study of 200 Patients

**DOI:** 10.1111/jop.70026

**Published:** 2025-08-11

**Authors:** Antonietta Argiuolo, Federica Canfora, Benedetta Muzii, Cristina D'Antonio, Daniela D'Auria, Amerigo Giudice, Gennaro Musella, Luca D'Aniello, Massimo Aria, Nelson Mauro Maldonato, Michele Davide Mignogna, Daniela Adamo

**Affiliations:** ^1^ Intradepartmental Program of Clinical Psychopathology Federico II University Hospital Naples Italy; ^2^ Department of Neurosciences, Reproductive Sciences and Odontostomatology University of Naples “Federico II” Naples Italy; ^3^ Department of Health Sciences, School of Dentistry University Magna Graecia of Catanzaro Catanzaro Italy; ^4^ Department of Clinical and Experimental Medicine University of Foggia Foggia Italy; ^5^ Department of Economics and Statistics University of Naples “Federico II” Naples Italy; ^6^ Department of Life Science, Health, and Health Professions Link Campus University Rome Italy

**Keywords:** anxiety, burning mouth syndrome, clinical global impression, depression, pain, sleep disturbance

## Abstract

**Background:**

This study investigates how anxiety, depression, and sleep disturbances affect pain perception and clinical impairment in burning mouth syndrome (BMS).

**Methods:**

A cross‐sectional survey was conducted on 200 BMS patients. The Hamilton Rating Scale for Depression (HAM‐D) and Anxiety (HAM‐A), Epworth Sleepiness Scale (ESS), Pittsburgh Sleep Quality Index (PSQI), Visual Analogue Scale (VAS), and short form of McGill pain questionnaire (SF‐MPQ) were used. The Clinical Global Impressions Severity of Illness (CGI‐S) assessed illness severity, and comorbidities were analyzed via the Age‐Adjusted Charlson Comorbidity Index (AACCI). Correlation tests and path analyses explored relationships among psychological factors, sleep quality, and BMS severity.

**Results:**

Pain intensity (VAS) correlated significantly with anxiety (HAM‐A, *ρ* = 0.25, *p* < 0.05), depression (HAM‐D, *ρ* = 0.15, *p* < 0.05), and shorter sleep duration (*ρ* = −0.19, *p* < 0.05). Path analyses revealed that anxiety significantly increased pain intensity (*β* = 0.24, *p* < 0.05), indirectly influencing clinical severity (CGI‐S: *β* = 0.07, *p* < 0.05). Depression strongly impacted poor sleep quality (PSQI, *β* = 0.33, *p* < 0.05). Shorter sleep duration affected both sleep quality (*β* = −0.46, *p* < 0.05) and clinical outcomes (CGI‐S, *β* = −0.17, *p* < 0.05). Pain quality (SF‐MPQ) showed weaker, non‐significant associations with psychological factors.

**Conclusions:**

Anxiety amplifies pain intensity, while depression worsens sleep quality, exacerbating clinical outcomes. Shorter sleep duration further contributes to worse outcomes. These findings emphasize the need for tailored interventions targeting psychological distress and sleep disturbances to improve pain management and quality of life in BMS patients.

## 1 Introduction

Burning mouth syndrome (BMS) is a chronic, idiopathic orofacial pain disorder characterized by a persistent burning sensation or pain in the oral mucosa, without identifiable local or systemic cause. Symptoms include xerostomia, altered taste, a foreign body sensation in the mouth, tingling, itching, and globus sensation, making it challenging to diagnose and manage [1]. Moreover, patients frequently report emotional distress and pain, with frustration and significant impairment in quality of life [2, 3].

The prevalence of BMS is estimated at 1.73% in the general population, with higher rates found among older adults, particularly postmenopausal women and dental patients [4, 5]. Indeed, BMS predominantly affects women, particularly in the perimenopausal and postmenopausal age ranges, with female‐to‐male ratios reported as high as 3:1 [4]. Sociodemographic and risk factors were found to differently influence pain perception in males and females affected by BMS. Therefore, clinicians should consider gender differences in the assessment of BMS patients to more effectively tailor pain management strategies [6].

Burning Mouth Syndrome (BMS) significantly impairs patient' quality of life, affecting essential functions such as eating, speaking, and sleeping [7]. Despite extensive research, BMS pathophysiology remains poorly understood. Current evidence suggests a multifactorial etiology involving both peripheral and central nervous system dysfunctions [8]. Importantly, psychological comorbidities–including anxiety, depression, sleep disturbances, and stress–are commonly observed in individuals with BMS and influence the severity and persistence of symptoms. Galli et al. [2] identified anxiety and depression as the most prevalent psychological factors associated with BMS, while Lee and colleagues (2023) [9] found that BMS patients have a significantly higher risk of developing both anxiety and depression, highlighting the strong bidirectional relationship between mental health and BMS. Anxiety and depression, in particular, are thought to exacerbate pain by lowering pain thresholds, intensifying perceived pain, and impairing natural pain modulation mechanisms [2, 10].

Recent epidemiological studies report that anxiety disorders are present in approximately 63.7% of BMS patients, while depressive disorders are observed to be present in around 36.3% of cases [11]. Moreover, a nationwide cohort study demonstrated that individuals with BMS have a higher risk of developing depression and anxiety compared to the general population [12]. These findings underscore the importance of addressing the psychological components of BMS in both research and clinical practice [13].

Additionally, sleep disturbances, commonly reported in BMS patients, exacerbate pain through a bidirectional cycle where poor sleep quality worsens anxiety and depression, which in turn amplify pain symptoms [14].

Given the multidimensional nature of BMS, understanding the intricate relationships between psychological factors, sleep disturbances, and pain perception is critical for optimizing patient care. However, few studies have specifically investigated the combined impact of anxiety, depression, and sleep disturbances on both patients' subjective pain experience and clinicians' assessment of disease severity in BMS. This study seeks to address these gaps. Its primary aim was to investigate the impact of anxiety, depression, and sleep disturbances on pain perception (intensity and quality) and clinical severity in BMS patients. It also explored the mediating role of sleep quality and the influence of age and sleep duration on clinical outcomes.

## 2 Materials and Methods

This study had a cross‐sectional design and was conducted retrospectively using data collected from medical records between 2022 and 2024 at the University of Naples “Federico II” Oral Medicine Department. All procedures followed the ethical principles of the Declaration of Helsinki and received approval from the local Ethical Committee (No. 251/19, 20/02/2019). The manuscript adheres to the STROBE reporting guidelines [15]. All participants provided written informed consent without compensation.

### 2.1 Participants

**FIGURE 1:**
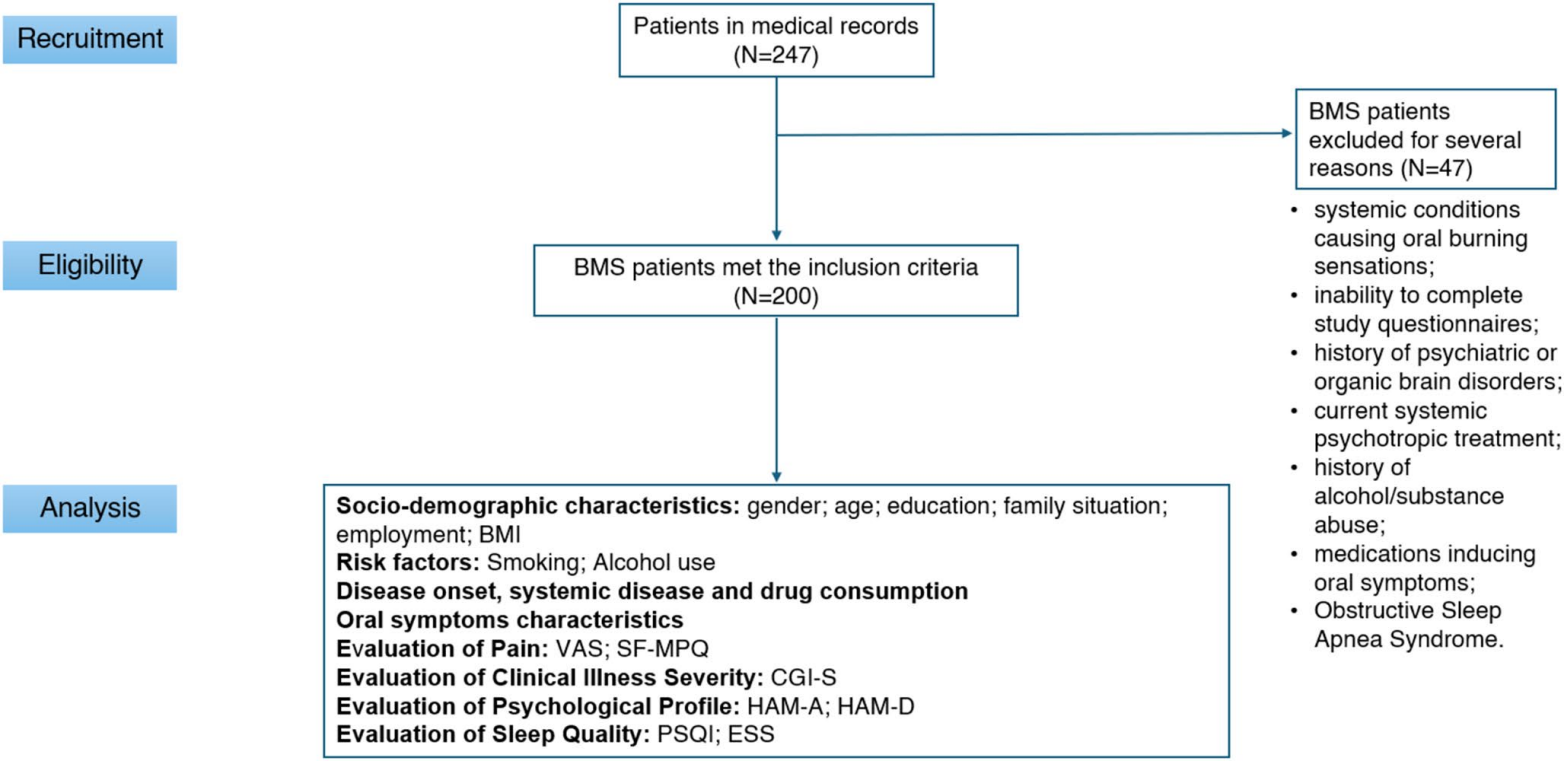
Flow chart of the current study. Abbreviations: BMI, Body Mass Index; BMS, burning mouth syndrome; CGI‐S, Clinical Global Impressions–Severity of Illness; ESS, Epworth Sleepiness Scale; HAM‐A, Hamilton Rating Scale for Anxiety; HAM‐D, Hamilton Rating Scale for Depression; PSQI, Pittsburgh Sleep Quality Index; SF‐MPQ, Short‐form McGill Pain Questionnaire; VAS, Visual Analogue Scale for pain intensity.

During the data collection period, a total of 247 patients with a diagnosis of BMS were inserted in the medical records of the Oral Medicine Unit. Among these, 200 met the inclusion criteria; thus, the study enrolled 200 BMS patients aged ≥ 18 years (Figure 1). All patients met International Classification of Orofacial Pain (2020) [16] criteria: intraoral burning or dysesthetic sensation recurring > 2 h daily for > 3 months, without evident causative lesions upon clinical examination.

As the ICOP classification does not differentiate between primary and secondary BMS, we included all patients meeting these diagnostic criteria as a single group, regardless of comorbid medical conditions or use of medications potentially associated with xerostomia. This approach reflects the current classificatory framework and supports a broader characterization of the BMS clinical phenotype. Participants exhibited normal laboratory parameters (complete blood count, fasting glucose, glycated hemoglobin, serum iron, ferritin, transferrin, folate, vitamin B12, and zinc) and were not receiving systemic psychotropic medications at enrollment.

All participants underwent a comprehensive oral examination by oral medicine specialists. Oral candidiasis was excluded based on the absence of clinical signs (e.g., white plaques, erythema, atrophic areas, and angular cheilitis). Patients with suspected candidiasis were excluded and referred for treatment. Oral hygiene was assessed clinically, and individuals with poor hygiene or signs of active periodontal or gingival disease were excluded to reduce potential confounding factors.

Exclusion criteria included:

- Systemic conditions causing oral burning sensations;
- Inability to complete study questionnaires;
- History of psychiatric or organic brain disorders;
- Current systemic psychotropic treatment;
- History of alcohol/substance abuse;
- Medications inducing oral symptoms as a direct and exclusive side effect in the absence of other criteria for BMS diagnosis;
- Obstructive Sleep Apnea Syndrome.

### 2.2 Data Collection and Measures

An oral medicine specialist (DA) performed intraoral and extraoral examinations for all participants. Data collection included demographics (age, gender, BMI, education, family status, and employment), risk factors (smoking, alcohol consumption), comorbidities, and current medications. Oral symptoms were comprehensively assessed, including intensity, diurnal patterns, and meal‐related improvements. All clinical assessments and questionnaires were administered during the patient' first visit to the Oral Medicine Unit. As this was a retrospective study, all data were extracted from medical records and reflect the patient' condition at intake.

The Age‐Adjusted Charlson Comorbidity Index (AACCI) [17], a well‐established tool that predicts 10‐year mortality based on a range of comorbidities, was used to assess comorbidity burden.

Patients with a known diagnosis of OSAS or those identified as at risk based on elevated scores on the ESS and PSQI were further evaluated with polysomnography; those with confirmed OSAS were excluded from the study.

### 2.3 Pain and Psychological Assessment

Pain and psychological assessment employed multiple validated instruments: Pain intensity was measured using the Visual Analogue Scale (VAS), a widely validated unidimensional tool in which patients indicated their pain level along a 10 cm horizontal line.

To assess pain quality, the Short‐Form McGill Pain Questionnaire (SF‐MPQ) [18] was employed. It captures various aspects of the pain experience, including sensory, affective, and evaluative dimensions. For all analyses, the total score was calculated as the sum of the 15 items (range: 0–45), with higher scores indicating more severe pain.

Depressive symptoms were assessed with the Hamilton Depression Rating Scale (HAM‐D) [19], a clinician‐administered tool based on an unstructured interview.

Anxiety was measured using the Hamilton Anxiety Rating Scale (HAM‐A) [20], which evaluates physical and psychological anxiety symptoms.

Symptoms of anxiety and depression were assessed using the HAM‐A and the HAM‐D, which evaluate symptom severity rather than establishing a formal diagnosis. All participants also underwent a psychiatric evaluation, and clinical diagnoses of anxiety and/or depression were confirmed by a psychiatrist when present.

Sleep quality was assessed using the Pittsburgh Sleep Quality Index (PSQI) [21], a self‐reported questionnaire, which assesses various dimensions of sleep over the past month. Moreover, the Epworth Sleepiness Scale (ESS) [22] measures daytime sleepiness, with ranges from 0 to 24. Additionally, subjective sleep hours, sleep disturbances before BMS diagnosis, and their duration in years were recorded.

Illness severity was assessed with the Clinical Global Impressions Severity of Illness (CGI‐S) [23] score. It is part of the clinical global impression scale and provides a standardized method for evaluating how ill a patient appears at a specific point in time. A clinician rated the overall impairment and gravity of the BMS on a scale from 1 to 7 (1 = not ill at all; 7 = among the most extremely ill patients).

### 2.4 Statistical Analyses

Statistical analyses were conducted using Jamovi (version 2.5.4; The jamovi project, 2022).

Descriptive statistics were calculated for all variables, with means, medians, and standard deviations for continuous data, and frequencies and percentages for categorical data. The Shapiro‑Wilk test assessed normality; non‐parametric tests were used as needed. We chose to report medians and interquartile ranges rather than means and standard deviations because several of the psychological variables (e.g., anxiety, depression, and sleep quality) exhibited significant deviations from normality, with skewed distributions. To ensure consistency and facilitate comparisons across measures, we reported medians for all variables.

Path analyses were employed to explore relationships between psychological factors (anxiety, depression), sleep disturbances, and how they contribute to perceived illness severity through pain outcomes (intensity and quality). Two models were developed: one for pain intensity (VAS) and one for pain quality (SF‐MPQ), incorporating sleep quality (PSQI), clinical severity (CGI‐S), and daytime sleepiness (ESS) as outcome variables, and anxiety (HAM‐A), depression (HAM‐D), sleep duration (Sleep‐h), and age as predictor variables. In both models, pain (either intensity or quality) is included as a mediator between psychological distress and clinical severity. Thus, the Clinical Global Impressions Severity Scale (CGI‐S) represents the final outcome of both models.

A bootstrap resampling technique (5000 iterations) ensured robust estimation, yielding standard errors, *p*‐values, and bias‐corrected 95% confidence intervals. Model fit was assessed using several fit indices, including the chi‐square test, Comparative Fit Index (CFI), Tucker‑Lewis Index (TLI), Root Mean Square Error of Approximation (RMSEA), and Standardized Root Mean Square Residual (SRMR). Final models are reported based on these indices.

All statistical tests were two‐tailed. For the correlation matrix, unadjusted *p*‐values are reported given the exploratory nature of the analysis. In path analyses, all coefficients were estimated simultaneously; no correction for multiple comparisons was thus needed.

### 2.5 Power Analysis

As this was a retrospective study, no a priori sample size calculation was performed. However, to evaluate the adequacy of our sample, we conducted a post hoc power analysis using GPower (version 3.1.9). For a multiple regression with four predictors and a medium effect size (*f* ^2^ = 0.15, *α* = 0.05), the achieved power with *N* = 200 was 0.99. These results indicate that the study was sufficiently powered to detect medium‐sized effects in the final step of the path model.

## 3 Results

### 3.1 Descriptive Statistics

Table 1 summarizes the sociodemographic profile, risk factors, systemic comorbidities, and medication use in 200 BMS patients. The demographic characteristics of our sample, predominantly female (159; 79.5%) with a median age of 68 years (IQR = 18), are consistent with previously published studies on BMS, which typically report a higher prevalence in postmenopausal women and a similar age distribution [24].

**TABLE 1:** Population demographics, comorbidities, and medication use in 200 BMS patients.

Demographic variables	Statistics	
	Median (IQR)	Range (Min–Max)
Age (in years)	64 (18)	21–85
Education (in years)	8 (7)	3–19
Body Mass Index (BMI)	26 (5.36)	17.7–45.4

	Frequency (%)
Gender	
Female Male	159 (79.5) 41 (20.5)
Family situation	
Married Widowed Divorced Single	143 (71.5) 24 (12) 17 (8.5) 16 (8)
Employment status	
Unemployed Employed Retired	88 (44) 56 (28) 56 (28)

Risk factors	Frequency (%)
Smoking (yes)	64 (32)
Alcohol use (yes)	43 (21.5)

Systemic comorbidities	Frequency (%)
Cardiovascular diseases	155 (77.5)
Metabilic/endocrine disorders	120 (60)
Gastrointestinal/hepatic issues	46 (23)
Oncological diseases	24 (13.5)
Respiratory diseases	13 (6.5)
Others	51 (25.5)

Drugs consumption	Frequency (%)
Antihypertensive/cardiovascular agents	134 (67)
Lipid‐lowering agents	95 (47.5)
Antithrombotic agents	62 (31)
Gastroprotective agents	53 (26.5)
Thyroid agents	30 (15)
Steroids	9 (4.5)
Osteoporosis medication	4 (2)
Other	42 (21)

Age‐Adjusted Charlson Comorbidity Index (AACCI)	Median (IQR)	Range (Min–Max)
	2 (2.25)	0–9

Regarding systemic comorbidities, 90% of the sample (*N* = 180) presented with at least one condition. The most prevalent category was cardiovascular diseases, reported by 77.5% of participants; within this group, hypertension was the most frequent condition (55%), followed by hypercholesterolemia (50.5%). Metabolic/endocrine disorders were also common (60%), particularly hypothyroidism (15%). The median AACCI score was 2, indicating a moderate comorbidity burden. The observed prevalence of systemic comorbidities—particularly cardiovascular and metabolic/endocrine conditions—is consistent with the expected profile for an older, predominantly female BMS population. Moreover, the tongue being the most commonly affected site aligns with prior studies identifying it as the most frequent location of BMS‐related symptoms [25, 26].

Table 2 highlights the oral and psychological symptom profile: all patients reported burning sensations, with 81% (*N* = 161) experiencing diffuse symptoms across the oral mucosa, and the tongue being the most affected site (*N* = 188; 94%) aligning with prior studies identifying it as the most frequent location of BMS‐related symptoms [27].

**TABLE 2:** Clinical and psychological features of BMS patients.

Oral symptoms	Frequency (%)
Burning	200 (100)
Xerostomia	147 (73.5)
Dysgeusia	100 (50)
Globus pharyngeus	82 (41)
Oral dysmorphism	77 (38.5)
Sialorrhea	48 (24)
Intraoral foreign body sensation	32 (16)
Oral dyskinesia	22 (11)
Tingling sensation	22 (11)
Occlusal dysesthesia	19 (9.5)
Burdening pain	17 (8.5)
Itching	14 (7)
Hypoesthesia	13 (6.5)
Dysosmia	13 (6.5)
Allodynia	8 (4)
Subjective halitosis	6 (3)

Location of pain/burning	Frequency (%)
Tongue	188 (94)
Palate	126 (63)
Lips	117 (58.5)
Gums	110 (55)
Perioral mucosa	99 (49.5)
Buccal mucosa	99 (49.5)
Floor of the mouth	98 (49)
Retromolar trigone	93 (46.5)

Pattern of symptoms	Frequency (%)
Continuous	96 (48)
Intermittent	82 (41)

Improving during meal	Frequency (%)
Yes	72 (36)
No	128 (64)

Pain evaluation	Median (IQR)	Range (Min–Max)
VAS	10 (2)	2–10
SF‐MPQ	12 (10.3)	1–42

Psychological profile	Median (IQR)	Range (Min–Max)
HAM‐A	16.5 (6)	7–35
HAM‐D	16 (6)	6–35

Sleep evaluation	Median (IQR)	Range (Min–Max)
Sleep duration (in hours)	6 (2)	3–10
PSQI total score	8 (4)	1–19
ESS	5 (4.25)	0–24
Sleep disturbances onset prior to BMS diagnosis (in years)	6 (6)	1–30

Clinical evaluation of severity of illness	Median (IQR)	Range (Min–Max)
CGI‐S	4 (1)	1–6

Abbreviations: BMS, Burning Mouth Syndrome; CGI‐S, Clinical Global Impressions–Severity of Illness; ESS, Epworth Sleepiness Scale; HAM‐A, Hamilton Rating Scale for Anxiety; HAM‐D, Hamilton Rating Scale for Depression; PSQI, Pittsburgh Sleep Quality Index; SF‐MPQ, Short‐form McGill Pain Questionnaire; VAS, Visual Analogue Scale.

Regarding pain, the median VAS score was 10 (IQR = 2), indicating very high pain intensity. The SF‐MPQ score was 12 (IQR = 10.3), consistent with moderate sensory‐affective pain experience.

Symptoms of psychological distress were prevalent in the sample. The median HAM‐A score was 16.5, and the HAM‐D was 16. Based on established cutoffs, 41.5% and 36% of participants, respectively, showed moderate‐to‐severe levels of anxiety and depressive symptoms. While these findings do not represent clinical diagnoses, they highlight a considerable emotional burden within this BMS cohort.

Sleep disturbances were also highly prevalent, since the median PSQI score of 8 (IQR: 4) indicated poor sleep quality in 89% (*N* = 179) of patients, with a median sleep duration of 6 h and below the recommended duration for healthy adults. The CGI‐S scale showed a median score of 4, reflecting moderate illness severity.

To explore preliminary associations among clinical and psychological variables, we generated a correlation matrix (Table A1). This analysis was intended to identify meaningful relationships that could inform and justify the structure of the subsequent path models. Anxiety (HAM‐A) and depression (HAM‐D) were strongly correlated (*ρ* = 0.64, *p* < 0.050), as expected from current scientific literature. Both HAM‐A (*ρ* = 0.33, *p* < 0.05) and HAM‐D (*ρ* = 0.4, *p* < 0.05) were associated with poor sleep quality (higher PSQI) and shorter sleep duration (*ρ* = −0.28, *p* < 0.05, and *ρ* = −0.22, *p* < 0.05).

Weak positive associations emerged between anxiety and pain intensity (VAS: *ρ* = 0.25, *p* < 0.05) and between depression and VAS (*ρ* = 0.15, *p* < 0.05), while anxiety also showed a small correlation with pain quality (SF‐MPQ: *ρ* = 0.14, *p* < 0.05). Poorer sleep quality (PSQI) was modestly associated with higher comorbidity burden (AACCI: *ρ* = 0.26, *p* < 0.05). Finally, CGI‐S was moderately and positively associated with VAS (*ρ* = 0.39, *p* < 0.05), HAM‐A (*ρ* = 0.32, *p* < 0.05), HAM‐D (*ρ* = 0.27, *p* < 0.05), and PSQI (*ρ* = 0.26, *p* < 0.05), while it was negatively associated with sleep duration (*ρ* = −0.27, *p* < 0.05), suggesting that more severely affected patients also report more psychological and somatic distress.

Given the conceptual differences between pain intensity and quality, and the observed lack of correlation between the two measures in our sample (*ρ* = 0.09, *p* > 0.05), we decided to explore them in two distinct path analyses models: one using VAS scores for pain intensity, and one using SF‐MPQ scores for pain quality.

### 3.2 Path Analysis With Pain Intensity (VAS): Model 1

The first path analysis model assessed how psychological distress, age, and sleep variables (HAM‐A, HAM‐D, Sleep‐h, Age, PSQI, and ESS) influenced illness severity (CGI‐S) through pain intensity (VAS) included as a mediator.

The model demonstrated excellent fit (χ^2^ (8) = 8.79, *p* > 0.05, Table 3). Variance explained by outcome variables ranged from moderate to minimal: PSQI (48.2%), CGI‐S (16%), VAS (6%), and ESS (3.1%).

**TABLE 3:** Fit indices and R‐squared values for path analysis model 1 with pain intensity and model 2 with pain quality.

Model 1		*χ* ^*2*^	df	*p*
Model tests	User model	8.79	8	0.36
	Baseline model	195.31	22	< 0.05
Fit indices	CFI	TLI	RMSEA	SRMR
	0.99	0.99	0.02	0.03
			**95% C. I.**	
	**Variable**	**R** ^**2**^	**Lower**	**Upper**
	PSQI	0.48	0.38	0.58
	CGI‐S	0.16	0.01	0.14
	VAS	0.06	0.07	0.26
	ESS	0.03	0.00	0.09

Model 2		*χ* ^2^	df	*p*
Model tests	User model	20	8	< 0.05
	Baseline model	180.2	22	< 0.05
Fit indices	CFI	TLI	RMSEA	SRMR
	0.92	0.79	0.09	0.04
			**95% C. I.**	
	**Variable**	**R** ^**2**^	**Lower**	**Upper**
	PSQI	0.48	0.38	0.58
	CGI‐S	0.09	0.03	0.18
	ESS	0.03	0.00	0.10
	SF‐MPQ	0.02	0.00	0.07

Abbreviations: CFI, Comparative Fit Index; C.I., Confidence Interval; CGI‐S, Clinical Global Impressions Severity of Illness; df, degrees of freedom; ESS, Epworth Sleepiness Scale; PSQI, Pittsburgh Sleep Quality Index; RMSEA, Root Mean Square Error of Approximation; SF‐MPQ, Short‐form McGill Pain Questionnaire; SRMR, Standardized Root Mean Square Residual; TLI, Tucker‐Lewis Index; VAS, Visual Analogue Scale for pain intensity.

**FIGURE 2:**
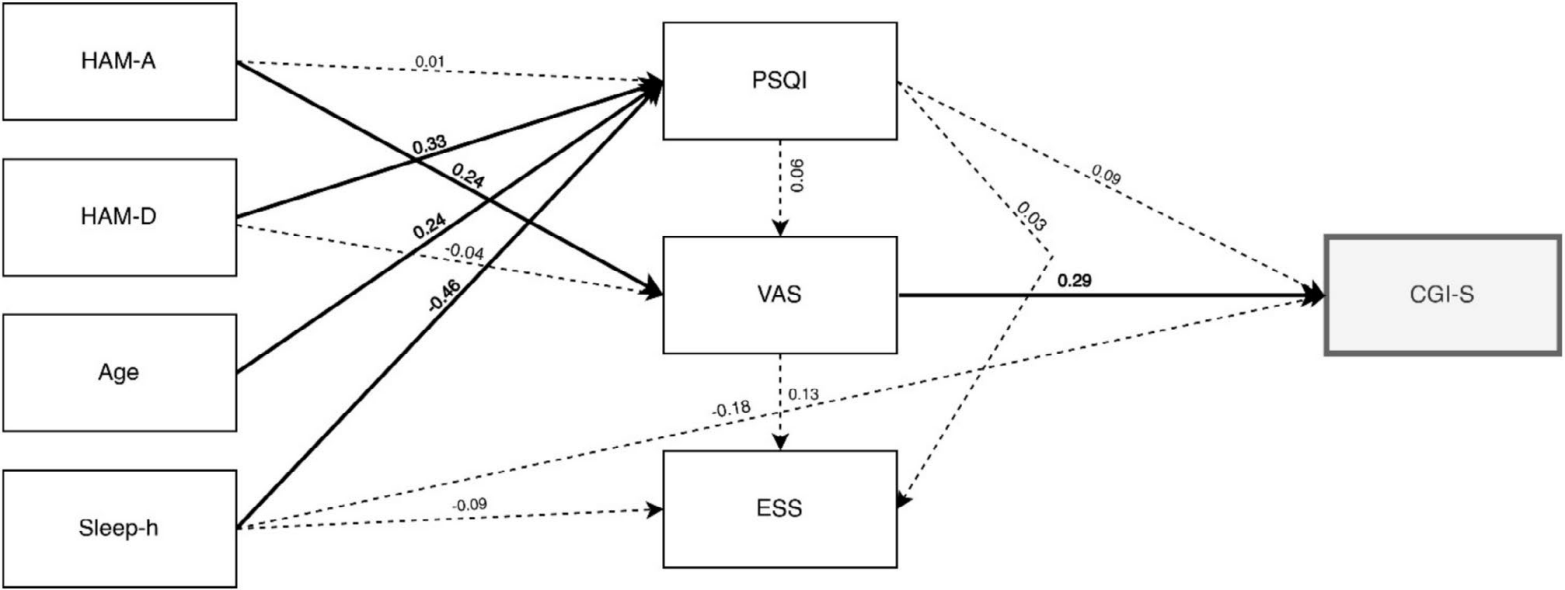
Path model showing standardized regression coefficients (*β*) with perceived clinical severity (CGI‐S) as the final outcome through pain intensity (VAS). Abbreviations: CGI‐S, Clinical Global Impressions Severity of Illness; ESS, Epworth Sleepiness Scale; HAM‐A, Hamilton Rating Scale for Anxiety; HAM‐D, Hamilton Rating Scale for Depression; PSQI, Pittsburgh Sleep Quality Index; VAS, Visual Analogue Scale for pain intensity. Significant paths and coefficients are shown in bold.

Key findings from the path analysis highlight that HAM‐A, HAM‐D, age, and sleep duration (Sleep‐h) have direct and indirect effects on PSQI, VAS, and CGI‐S (Table 4 and Figure 2).

**TABLE 4:** Path analysis model 1 (VAS) and 2 (SF‐MPQ).

Path analysis model 1: Direct effects on pain intensity (VAS)			95% C. I.			
Direct effects	Coefficient	SE	Lower	Upper	*β*	*p*
HAM‐A ⇒ PSQI	0.01	0.07	−0.11	0.11	0.01	0.93
HAM‐D ⇒ PSQI	0.23	0.05	0.13	0.34	0.33	< 0.05
Age ⇒ PSQI	0.07	0.01	0.04	0.10	0.24	< 0.05
Sleep‐h ⇒ PSQI	−1.27	0.15	−1.56	−0.96	−0.46	< 0.05
HAM‐A ⇒ VAS	0.07	0.03	0.02	0.12	0.24	< 0.05
HAM‐D ⇒ VAS	−0.01	0.03	−0.06	0.04	−0.04	0.68
PSQI ⇒ VAS	0.03	0.04	−0.05	0.09	0.06	0.50
PSQI ⇒ CGI‐S	0.02	0.01	−0.01	0.04	0.09	0.25
Sleep‐h ⇒ CGI‐S	−0.09	0.05	−0.17	0.01	−0.18	0.06
VAS ⇒ CGI‐S	0.12	0.03	0.06	0.19	0.29	< 0.05
PSQI ⇒ ESS	0.03	0.08	−0.12	0.18	0.03	0.67
Sleep‐h ⇒ ESS	−0.25	0.26	−0.77	0.25	−0.09	0.34
VAS ⇒ ESS	0.33	0.17	−0.01	0.64	0.13	0.05

Path analysis model 2: Direct effects on pain quality (SF‐MPQ)			95% C. I.			
Direct effects	Coefficient	SE	Lower	Upper	*β*	*p*
HAM‐A ⇒ PSQI	0.01	0.06	−0.12	0.11	0.01	0.93
HAM‐D ⇒ PSQI	0.23	0.05	0.13	0.34	0.33	< 0.05
Age ⇒ PSQI	0.07	0.01	0.04	0.10	0.24	< 0.05
Sleep‐h ⇒ PSQI	−1.27	0.15	−1.56	−0.96	−0.46	< 0.05
HAM‐A ⇒ SF‐MPQ	0.20	0.16	−0.10	0.52	0.11	0.22
HAM‐D ⇒ TPRI	−0.07	0.16	−0.40	0.23	−0.04	0.65
PSQI ⇒ SF‐MPQ	0.19	0.18	−0.17	0.55	0.08	0.30
PSQI ⇒ CGI‐S	0.02	0.02	−0.01	0.05	0.09	0.28
Sleep‐h ⇒ CGI‐S	−0.12	0.05	−0.21	−0.01	−0.23	< 0.05
SF‐MPQ ⇒ CGI‐S	0.00	0.01	−0.01	0.01	0.04	0.48
PSQI ⇒ ESS	0.02	0.08	−0.14	0.16	0.02	0.84
Sleep‐h ⇒ ESS	−0.37	0.26	−0.89	0.11	−0.13	0.16
SF‐MPQ ⇒ ESS	0.05	0.03	−0.02	0.12	0.11	0.14

*Note: p*‐values based on BCa bootstrapping (5,000 samples); two‐tailed.

Abbreviations: CGI‐S, Clinical Global Impressions Severity of Illness; C.I., Confidence Intervals; ESS, Epworth Sleepiness Scale; HAM‐A, Hamilton Rating Scale for Anxiety; HAM‐D, Hamilton Rating Scale for Depression; SE, Standard Error; SF‐MPQ, Short‐form McGill Pain Questionnaire; Sleep‐h, hours of sleep; PSQI, Pittsburgh Sleep Quality Index; VAS, pain intensity as measured with the Visual Analogue Scale.

Direct Effects:

- Depression and Sleep Quality: Higher levels of depression were associated with poorer sleep quality (HAM‐D → PSQI: *β* = 0.33, *p* < 0.05, 95% CI = [0.13, 0.34]).
- Age and Sleep Quality: Older participants reported worse sleep quality (Age → PSQI: *β* = 0.24, *p* < 0.05, 95% CI = [0.04, 0.10]).
- Sleep Duration and Sleep Quality: Fewer hours of sleep were linked to poorer sleep quality (Sleep‐h → PSQI: *β* = −0.46, *p* < 0.05, 95% CI = [−1.56, −0.96]).
- Anxiety and Pain Intensity: Higher anxiety was related to increased pain intensity (HAM‐A → VAS: *β* = 0.24, *p* < 0.05, 95% CI = [0.02, 0.12]).
- Pain Intensity and Illness Severity: Greater pain intensity was associated with higher clinical severity (VAS → CGI‐S: *β* = 0.29, *p* < 0.05, 95% CI = [0.06, 0.19]).

Indirect Effects (for a complete list of indirect effect, see Table A2):

- Anxiety and Illness Severity via Pain: Anxiety had a significant indirect effect on clinical severity, mediated by pain intensity (HAM‐A → VAS → CGI‐S: *β* = 0.07, *p* < 0.05, 95% CI = [0.00, 0.02]).
- Age and Illness Severity via Sleep Quality: no significant indirect effect of Age on CGI‐S via PSQI was revealed (*p* > 0.05).

### 3.3 Path Analysis With Pain Quality (SF‐MPQ): Model 2

**FIGURE 3:**
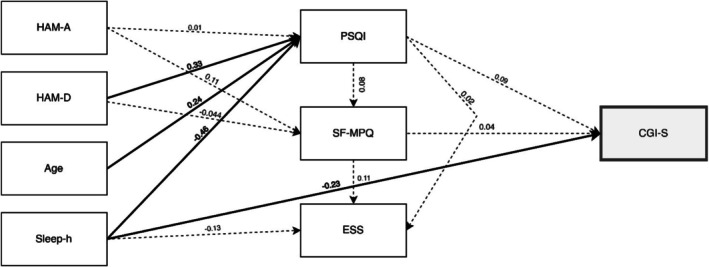
Path model showing standardized regression coefficients (*β*) with perceived clinical severity (CGI‐S) as the final outcome through pain quality (total SF‐MPQ score). Abbreviations: CGI‐S, Clinical Global Impressions Severity of Illness; ESS, Epworth Sleepiness Scale; HAM‐A, Hamilton Rating Scale for Anxiety; HAM‐D, Hamilton Rating Scale for Depression; PSQI, Pittsburgh Sleep Quality Index; SF‐MPQ, Short‐form McGill Pain Questionnaire. Significant paths and coefficients are shown in bold.

The second path analysis model assessed how anxiety (HAM‐A), depression (HAM‐D), sleep duration (Sleep‐h), and age influence perceived illness severity (CGI‐S) through pain quality (SF‐MPQ), calculated as the sum of the SF‐MPQ items (Table 4 and Figure 3).

The model showed acceptable fit, slightly weaker than the first (χ^2^ (8) = 20, *p* < 0.05, Table 3).

Variance explained (R^2^) was lower: PSQI (48%), CGI‐S (9%), SF‐MPQ (2%), ESS (3%). Results reveal direct effects of HAM‐D, Sleep‐h, and Age on PSQI, as in the first model (Table 4). Moreover, there was a direct effect of Sleep‐h on CGI‐S: longer sleep reduces illness severity (*β* = −0.23, *p* < 0.05, 95% CI = [−0.21, −0.01]). No significant indirect effects were revealed by this model (see Table A3). Notably, this included the indirect relationship between Age and CGI‐S (via PSQI through SF‐MPQ) which did not reach statistical significance. Although no control group was included in this study, the observed association between reduced sleep duration and greater illness severity is consistent with findings in other chronic pain and sleep disorder populations, where poorer sleep is linked to elevated CGI‐S scores [27, 28].

Our findings show a direct effect of sleep duration on perceived illness severity, suggesting that reduced sleep independently contributes to worse clinical impressions in BMS patients. This is consistent with evidence that sleep disturbances not only exacerbate pain and mood symptoms but also increase functional impairment in chronic pain disorders. Notably, age also exerted a direct effect on sleep quality (PSQI), which aligns with prior studies indicating that aging is a risk factor for sleep fragmentation and reduced sleep efficiency [29].

In the context of BMS, this is particularly relevant, as older adults are disproportionately affected by both sleep disorders and BMS itself. Previous research has highlighted the bidirectional relationship between poor sleep and chronic pain, as well as the mediating role of psychological distress in this interplay. Our model adds to this literature by demonstrating that while age and depression affect sleep, only sleep duration had a direct effect on perceived illness severity (CGI‐S), underscoring its potential role as a modifiable target in BMS management.

## 4 Discussion

This study underscores the multifactorial nature of BMS, emphasizing the critical roles of anxiety, depression, and sleep disturbances in shaping pain perception and clinical severity. These findings align with previous research demonstrating that these factors not only frequently co‐occur in BMS patients but also exacerbate pain intensity and contribute to the perception of clinical impairment [2, 30]. A key strength of this study lies in its detailed exploration of these components and their interconnections.

In this study, BMS patients reported high levels of pain severity, reaffirming its role as a central and debilitating feature of the syndrome. Nearly all patients (99%) experienced anxiety and depression, and sleep quality was significantly impaired in 89% of patients. Additionally, 60% of patients reported sleep disturbances with a median onset 6 years before their BMS diagnosis, suggesting that sleep disorders may predispose individuals to or exacerbate BMS symptoms. Clinical severity, as measured by the CGI‐S score, reflected a moderate‐to‐severe disease burden.

The CGI‐S scores observed in our cohort are consistent with prior findings in populations affected by chronic pain, sleep disorders, and BMS, typically ranging from 4 to 5, while healthy controls usually score 1, indicating no perceived illness.

The correlation analysis emphasized the strong link between anxiety and depression, which often co‐occur in BMS. Both conditions were also significantly related to poor sleep quality, suggesting a reinforcing cycle between psychological distress and sleep impairments that likely worsens symptoms. Pain intensity showed a moderate association with both anxiety and depression, highlighting the amplifying effect of psychological factors on pain perception. Demographic factors such as age and education also affected sleep quality and psychological burden, with older patients and those with lower education levels experiencing worse outcomes.

Although anxiety and depression are strongly associated with both poor sleep quality and pain intensity, the path analysis provided a deeper understanding of their distinct pathways in influencing illness severity. Anxiety directly increased pain intensity, which in turn indirectly influenced illness severity as assessed by CGI‐S. Depression, by contrast, had a more direct impact on sleep quality, reinforcing its role in shaping clinical outcomes through indirect mechanisms. These findings underscore the limitations of correlation analyses and highlight the value of path analysis in disentangling the distinct pathways through which psychological distress, sleep disturbances and pain affect BMS outcomes.

The path analyses results confirm the central role of anxiety in amplifying pain intensity and driving symptom burden in BMS patients [10].

The association between anxiety and pain intensity in BMS has been documented in previous studies. For instance, Amenabar et al. [31] identified salivary cortisol as a potential marker of stress‐related pain exacerbation, while Porporatti et al. [32] reported links between psychological distress and increased nociceptive sensitivity, mediated by activation of the hypothalamic‐pituitary‐adrenal (HPA) axis and inflammatory responses [33, 34]. However, in our study, although anxiety levels were significantly associated with higher self‐reported pain intensity, no significant relationship was observed between anxiety and pain quality [35, 36]. This suggests that anxiety may more strongly influence the intensity rather than the qualitative dimensions of pain [37].

Our findings are particularly relevant when considered within the broader context of age, sleep quality and pain in women. Prior research has shown that advancing age and female sex are associated with altered sleep architecture, increased prevalence of sleep disturbances and heightened pain sensitivity. Poor sleep quality has also been linked to central sensitization and reduced pain thresholds, particularly in women [38, 39]. These interactions likely contribute to the complex relationship observed in our study between psychological distress, sleep disruption and pain perception. Further research is warranted to clarify how these variables interact over time and influence clinical outcomes in patients with chronic oral symptoms.

In addition, this study highlighted the pivotal role of sleep quality in shaping clinical outcomes. Although depression did not directly influence pain intensity, it emerged as a strong predictor of poor sleep quality, which in turn aggravated subjective pain perception and clinician‐assessed impairment. These findings are consistent with existing literature on the bidirectional relationship between sleep disruptions and depression [40]. Sleep disturbances are symptoms of depression and significant risk factors for its onset [41], suggesting that improving sleep quality could have positive effects on both depression and pain outcomes in BMS. Age and sleep duration were also significant predictors, with older patients reporting poorer sleep. However, sleep disturbances did not directly affect pain intensity; further emphasizing the distinct pathways through which sleep and psychological distress impact BMS outcomes. Notably, the differing impacts of anxiety and depression observed in this model suggest that these psychological factors may differentially affect various aspects of BMS.

The path analysis of Model 2 demonstrated that anxiety and depression significantly impacted sleep quality, with shorter sleep duration strongly associated with poorer sleep quality. However, neither anxiety, depression, nor sleep quality had a significant direct effect on pain quality; suggesting that unmeasured factors may mediate the relationship between psychological distress, sleep disturbances, and the qualitative aspects of pain.

While Model 2 offered valuable insights, it did not substantially improve our understanding of BMS outcomes compared to Model 1, which provides a clearer and more actionable explanation of the interplay between anxiety, depression, sleep disturbances, and clinical severity, emphasizing the need for complementary pain measures to fully capture the multidimensional nature of BMS symptoms.

From a methodological standpoint, it is important to acknowledge the potential complexity of assessing pain quality within our study sample. The Italian translation of the SF‐MPQ includes descriptors such as “splitting pain” or “stabbing pain,” which can be challenging for participants to interpret, regardless of their education level. Moreover, considering the high median age of our sample (64 years) and the historical context—in Italy, only about 30% of adolescents accessed high school fifty years ago—lower educational attainment is common among this age group and reflects broader social trends rather than individual limitations.

The absence of a significant correlation between pain intensity and pain quality in our sample was unexpected, as these dimensions are often moderately related in chronic pain populations. While previous research has reported mixed findings—with some studies showing significant correlations [6], and others not [42] this divergence in our data led us to model pain intensity (VAS) and pain quality (SF‐MPQ) separately. This decision was not only data‐driven but also grounded in theory, considering that pain intensity reflects the magnitude of pain, whereas pain quality captures its qualitative and affective characteristics [43, 44, 45]. Notably, in our models, anxiety directly predicted pain intensity (VAS), which in turn significantly predicted overall clinical severity (CGI‐S), whereas no significant predictors or downstream effects were observed for pain quality (SF‐MPQ). These findings suggest that intensity and quality may be partially independent dimensions of the pain experience, differentially shaped by psychological variables and with distinct implications for clinical status.

Notably, in both models, depression does not significantly influence pain; despite the significant correlation between HAM‐D scores and VAS scores. This suggests that the relationship between depression and pain is likely mediated by other factors rather than being a straightforward causal link. Shared biological mechanisms, including neurotransmitter dysregulations (serotonin, dopamine, and norepinephrine) and heightened inflammatory responses, may contribute to the complex interaction between the two [46].

Furthermore, it remains unclear whether psychopathological factors, such as anxiety and depression, act as causes or consequences of BMS [2].

Our findings refine previous literature by identifying anxiety as a direct contributor to pain intensity and depression as a key factor in poor sleep quality, both of which influence clinical severity in BMS. Unlike earlier studies that found no significant differences in anxiety between BMS patients and controls, our results suggest a stronger role for anxiety when assessed dimensionally [47, 48].

Additionally, we highlight reduced sleep duration as a distinct mediator of worse clinical outcomes, expanding on prior work that broadly reported poor sleep quality. These differences emphasize the need for targeted psychological and sleep‐related interventions in BMS management.

The lack of a significant predictive relationship between sleep and pain warrants further investigation, considering methodological factors and alternative theoretical explanations. This study highlights the necessity of a multidimensional biopsychosocial approach to BMS management, as anxiety amplifies pain intensity—likely via central sensitization and hypervigilance—while depression primarily impairs sleep quality, exacerbating subjective pain perception. However, the cross‐sectional design precludes causal inferences, and reliance on self‐reported measures may introduce recall bias; future research should incorporate longitudinal designs and objective assessments such as neuroimaging, polysomnography, and inflammatory biomarkers. The sample's demographic homogeneity limits generalizability, and unaccounted factors like medication use and hormonal influences may have influenced results. Additionally, the linear framework of path analysis may not fully capture BMS complexity.

A significant limitation of this study is the reliance on self‐reported measures to assess pain intensity, sleep quality, and psychological symptoms. While these instruments are widely used and validated, they are inherently subject to several biases, such as accuracy in reporting the frequency or severity of symptoms and social desirability, that might have led some individuals to underreport emotional distress or overstate their psychological resilience. These biases may limit the precision of the data and should be considered when interpreting the results.

Another limitation concerns the lack of information on potential confounding variables such as dietary habits, physical activity, and socioeconomic status. These factors are known to influence psychological well‐being and sleep quality and could have impacted the associations observed in this study. Future prospective studies should consider including these variables to more accurately assess their contribution and potential confounding role in the relationships among psychological symptoms, sleep disturbances, and BMS‐related pain.

Given the cross‐sectional nature of the present study, causal relationships between psychological factors and pain perception in BMS cannot be inferred. Longitudinal studies are needed to better clarify the temporal dynamics and potential bidirectionality of these associations, particularly in understanding whether psychological distress contributes to the onset or maintenance of BMS‐related pain over time.

Despite these limitations, the study provides valuable insights into the interplay of psychological factors, sleep disturbances, and pain, forming a basis for future research and treatment strategies.

As regards practical implications, our findings have relevant clinical suggestions for the management of BMS. The significant associations with anxiety and depression highlight the need for a more integrated diagnostic approach that considers both psychological and physical aspects. Incorporating psychological screening into routine assessment may improve diagnostic accuracy and guide targeted interventions.

Given the role of psychological distress in BMS, therapeutic strategies such as Cognitive Behavioral Therapy (CBT) may usefully complement standard treatments, improving symptom management and patients' quality of life. A multidisciplinary approach involving physicians, psychologists, and other healthcare providers is recommended to ensure comprehensive care.

Training clinicians to recognize and address psychological factors in BMS could facilitate earlier identification of at‐risk patients and more effective interventions. These findings may also inform the development of clinical guidelines that integrate psychological assessment and care, promoting more personalized and effective treatment pathways.

## 5 Conclusions

In conclusion, our study provides valuable insights into the complex relationship between psychological factors—such as anxiety, depression, and sleep disturbances—and pain perception in patients with BMS. The findings suggest that these psychological factors significantly impact the severity of BMS symptoms, underscoring the importance of a holistic approach in diagnosis and treatment.

Future research should also explore the efficacy of psychological interventions aimed at reducing anxiety, depression, and sleep disturbances in patients with BMS. Investigating whether targeted treatments—such as Cognitive Behavioral Therapy or mindfulness‐based approaches—can alleviate pain perception would provide valuable insights into the potential for integrated, multidisciplinary care strategies in this population.

## Author Contributions

**Antonietta Argiuolo:** investigation, writing – original draft. **Federica Canfora:** data curation, writing – original draft. **Benedetta Muzii:** writing – original draft. **Cristina D'Antonio:** investigation, visualization. **Daniela D'Auria:** investigation, data curation. **Amerigo Giudice:** methodology, data curation. **Gennaro Musella:** methodology, visualization, writing – original draft. **Luca D'Aniello:** statistical analysis, formal analysis; **Massimo Aria:** statistical analysis, formal analysis. **Nelson Mauro Maldonato:** conceptualization, writing – review and editing. **Michele Davide Mignogna:** conceptualization, validation. **Daniela Adamo:** conceptualization, supervision, writing – review and editing.

## Ethics Statement

This study was approved by the Ethical Committee (No. 251/19, 20/02/2019).

## Consent

Informed consent was obtained from all patients for this study.

## Conflicts of Interest

The authors declare no conflicts of interest.

## Peer Review

The peer review history for this article is available at https://www.webofscience.com/api/gateway/wos/peer‐review/10.1111/jop.70026.

## Supporting information


**Data S1:** STROBE Statement—Checklist of items that should be included in reports of *cross‐sectional studies*.

## Data Availability

The data that support the findings of this study are available from the corresponding author upon reasonable request.

## Appendix A

**TABLE A1:** Correlation matrix of clinical and psychological variables in BMS patients.

	Age	Education	BMI	Onset (months)	HAM‐A	HAM‐D	VAS	SF‐MPQ	Sleep (h)	PSQI	ESS	AACCI	CGI‐S
Age	—												
Education	‐0.25*	—											
BMI	0.15*	−0.17*	—										
Onset (months)	−0.03	−0.05	−0.01	—									
HAM‐A	0.06	−0.18*	0.10	0.06	—								
HAM‐D	0.05	−0.11	−0.01	0.01	0.64*	—							
VAS	−0.08	−0.03	0.10	0.02	0.25*	0.15*	—						
SF‐MPQ	−0.04	−0.08	0.06	0.10	0.14*	0.13	0.09	—					
Sleep (h)	−0.07	−0.05	−0.09	0.08	−0.28*	−0.22*	−0.19*	0.05	—				
PSQI	0.29*	−0.18*	0.17*	−0.01	0.33*	0.40*	0.12	0.13	−0.58*	—			
ESS	−0.02	0.02	0.07	−0.08	0.01	0.01	0.17*	0.06	−0.14	0.10	—		
AACCI	0.73*	−0.21*	0.17*	0.00	0.13	0.13	−0.08	0.02	−0.12	0.26*	−0.01	—	
CGI‐S	−0.03	−0.05	0.15*	−0.05	0.32*	0.27*	0.39*	0.05	−0.27*	0.26*	0.12	0.02	—

*Note*: **p* < 0.05.

Abbreviations: AACCI, Age‐Adjusted Charlson Comorbidity Index; BMI, Body Mass Index; CGI‐S, Clinical Global Impressions–Severity of Illness; ESS, Epworth Sleepiness Scale; HAM‐A, Hamilton Rating Scale for Anxiety; HAM‐D, Hamilton Rating Scale for Depression; SF‐MPQ, Short‐form McGill Pain Questionnaire; PSQI, Pittsburgh Sleep Quality Index; MPQ, Short‐form McGill Pain Questionnaire; VAS, Visual Analogue Scale.

**TABLE A2:** Indirect effects in path analysis model 1 (VAS).

			95% C. I.			
Indirect effects	Coefficient	SE	Lower	Upper	*β*	*p*
HAM‐A ⇒ PSQI ⇒ VAS ⇒ CGI‐S	0.00	0.00	0.00	0.00	0.00	0.96
HAM‐A ⇒ PSQI ⇒ VAS ⇒ ESS	0.00	0.00	0.00	0.00	0.00	0.96
HAM‐A ⇒ PSQI ⇒ CGI‐S	0.00	0.00	0.00	0.00	0.00	0.95
HAM‐A ⇒ PSQI ⇒ ESS	0.00	0.01	−0.01	0.01	0.00	0.97
HAM‐A ⇒ VAS ⇒ CGI‐S	0.01	0.00	0.00	0.02	0.07	< 0.05
HAM‐A ⇒ VAS ⇒ ESS	0.02	0.01	−0.01	0.05	0.03	0.09
HAM‐D ⇒ PSQI ⇒ VAS ⇒ CGI‐S	0.00	0.00	0.00	0.00	0.01	0.53
HAM‐D ⇒ PSQI ⇒ VAS ⇒ ESS	0.00	0.00	−0.01	0.01	0.00	0.56
HAM‐D ⇒ PSQI ⇒ CGI‐S	0.00	0.00	0.00	0.01	0.03	0.29
HAM‐D ⇒ PSQI ⇒ ESS	0.01	0.02	−0.03	0.05	0.01	0.66
HAM‐D ⇒ VAS ⇒ CGI‐S	0.00	0.00	−0.01	0.01	−0.01	0.69
HAM‐D ⇒ VAS ⇒ ESS	0.00	0.01	−0.02	0.02	0.00	0.73
Sleep‐h ⇒ PSQI ⇒ VAS ⇒ CGI‐S	0.00	0.01	−0.02	0.01	−0.01	0.51
Sleep‐h ⇒ PSQI ⇒ VAS ⇒ ESS	−0.01	0.02	−0.04	0.03	0.00	0.54
Sleep‐h ⇒ PSQI ⇒ CGI‐S	−0.02	0.02	−0.06	0.01	−0.04	0.26
Sleep‐h ⇒ PSQI ⇒ ESS	−0.04	0.10	−0.24	0.16	−0.02	0.67
Age ⇒ PSQI ⇒ VAS ⇒ CGI‐S	0.00	0.00	0.00	0.00	0.00	0.52
Age ⇒ PSQI ⇒ VAS ⇒ ESS	0.00	0.00	0.00	0.00	0.00	0.56
Age ⇒ PSQI ⇒ CGI‐S	0.00	0.00	0.00	0.00	0.02	0.27
Age ⇒ PSQI ⇒ ESS	0.00	0.01	−0.01	0.01	0.01	0.67
PSQI ⇒ VAS ⇒ CGI‐S	0.00	0.01	−0.01	0.01	0.02	0.51
PSQI ⇒ VAS ⇒ ESS	0.01	0.01	−0.02	0.03	0.01	0.54

*Note: p*‐values based on BCa bootstrapping (5,000 samples), two‐tailed.

Abbreviations: CGI‐S, Clinical Global Impressions Severity of Illness; C.I., Confidence Intervals; ESS, Epworth Sleepiness Scale; HAM‐A, Hamilton Rating Scale for Anxiety; HAM‐D, Hamilton Rating Scale for Depression; PSQI, Pittsburgh Sleep Quality Index; VAS, pain intensity as measured with the Visual Analogue Scale; SE, standard error; Sleep‐h, hours of sleep.

**TABLE A3:** Indirect effects in path analysis model 2 (SF‐MPQ).

			95% C. I.			
Indirect effects	Coefficient	SE	Lower	Upper	*β*	*p*
HAM‐A ⇒ PSQI ⇒ SF‐MPQ ⇒ CGI‐S	0.00	0.00	0.00	0.00	0.00	0.97
HAM‐A ⇒ PSQI ⇒ SF‐MPQ ⇒ ESS	0.00	0.00	0.00	0.00	0.00	0.96
HAM‐A ⇒ PSQI ⇒ CGI‐S	0.00	0.00	0.00	0.00	0.00	0.95
HAM‐A ⇒ PSQI ⇒ ESS	0.00	0.01	−0.01	0.01	0.00	0.99
HAM‐A ⇒ SF‐MPQ ⇒ CGI‐S	0.00	0.00	0.00	0.00	0.01	0.62
HAM‐A ⇒ SF‐MPQ ⇒ ESS	0.01	0.01	−0.01	0.03	0.01	0.37
HAM‐D ⇒ PSQI ⇒ SF‐MPQ ⇒ CGI‐S	0.00	0.00	0.00	0.00	0.00	0.65
HAM‐D ⇒ PSQI ⇒ SF‐MPQ ⇒ ESS	0.00	0.00	−0.01	0.01	0.00	0.48
HAM‐D ⇒ PSQI ⇒ CGI‐S	0.00	0.00	0.00	0.01	0.03	0.31
HAM‐D ⇒ PSQI ⇒ ESS	0.00	0.02	−0.03	0.04	0.01	0.85
HAM‐D ⇒ SF‐MPQ ⇒ CGI‐S	0.00	0.00	0.00	0.00	0.00	0.80
HAM‐D ⇒ SF‐MPQ ⇒ ESS	0.00	0.01	−0.02	0.02	−0.01	0.71
Age ⇒ PSQI ⇒ SF‐MPQ ⇒ CGI‐S	0.00	0.00	0.00	0.00	0.00	0.66
Age ⇒ PSQI ⇒ SF‐MPQ ⇒ ESS	0.00	0.00	0.00	0.00	0.00	0.47
Age ⇒ PSQI ⇒ CGI‐S	0.00	0.00	0.00	0.00	0.02	0.28
Age ⇒ PSQI ⇒ ESS	0.00	0.01	−0.01	0.01	0.00	0.85
Sleep‐h ⇒ PSQI ⇒ SF‐MPQ ⇒ CGI‐S	0.00	0.00	0.00	0.00	0.00	0.65
Sleep‐h ⇒ PSQI ⇒ SF‐MPQ ⇒ ESS	−0.01	0.02	−0.04	0.03	0.00	0.47
Sleep‐h ⇒ PSQI ⇒ CGI‐S	−0.02	0.02	−0.06	0.02	−0.04	0.28
Sleep‐h ⇒ PSQI ⇒ ESS	−0.02	0.10	−0.21	0.19	−0.01	0.85
PSQI ⇒ SF‐MPQ ⇒ CGI‐S	0.00	0.00	0.00	0.00	0.00	0.65
PSQI ⇒ SF‐MPQ ⇒ ESS	0.01	0.01	−0.02	0.03	0.01	0.47

*Note: p*‐values based on BCa bootstrapping (5,000 samples), two‐tailed.

Abbreviations: CGI‐S, Clinical Global Impressions Severity Of Illness; C.I., confidence intervals; ESS, Epworth Sleepiness Scale; HAM‐A, Hamilton Rating Scale for Anxiety; HAM‐D, Hamilton Rating Scale for Depression; PSQI, Pittsburgh Sleep Quality Index; SE, standard error; Sleep‐h, hours of sleep.
